# Temperature influences outcomes of an environmentally acquired symbiosis

**DOI:** 10.1093/ismejo/wraf056

**Published:** 2025-03-21

**Authors:** Patrick T Stillson, Kaisy Martinez, Johnathan Adamson, Arshya Tehrani, Alison Ravenscraft

**Affiliations:** Department of Biology, University of Texas at Arlington, 501 S Nedderman Drive, Arlington, TX 76019, United States; Department of Biology, Emory University, 1510 Clifton Road NE, Atlanta, GA 30322, United States; Department of Biology, University of Texas at Arlington, 501 S Nedderman Drive, Arlington, TX 76019, United States; Department of Biology, University of Texas at Arlington, 501 S Nedderman Drive, Arlington, TX 76019, United States; Department of Biology, University of Texas at Arlington, 501 S Nedderman Drive, Arlington, TX 76019, United States; Department of Biological Sciences, University of Texas at Dallas, 800 W Campbell Road, Richardson, TX 75080, United States; Department of Biology, University of Texas at Arlington, 501 S Nedderman Drive, Arlington, TX 76019, United States

**Keywords:** *Caballeronia*, context dependence, heteroptera, symbiosis, temperature, thermal response

## Abstract

Microbial symbioses are essential for many animals, but their outcomes are often context dependent. For example, rising temperatures can disrupt symbioses by eliminating thermally sensitive symbionts. The temperature tolerance of a symbiont may therefore limit the temperature range of its host, but switching to a more thermally tolerant partner could expand this range. Eastern leaf footed bugs (*Leptoglossus phyllopus*) depend on symbiotic *Caballeronia* bacteria which they must acquire from the environment early in development. Could this result in intergenerational partner switching that improves host outcomes under changing conditions? As a first step towards answering this question, we tested the hypothesis that host outcomes in this symbiosis vary among symbiont strains in a temperature-dependent manner. Nymphs were provided with one of six *Caballeronia* strains with varying thermal optima and reared at temperatures from 24–40°C. We observed temperature- and strain-dependent tradeoffs in host outcomes, with different strains conferring improved host weight, development time, and survival at cooler versus warmer temperatures. Differences in host outcomes were most pronounced at high temperatures, with some strains imposing severe costs. However, *Caballeronia*’s *in vitro* thermal optima did not predict *in vivo* outcomes. Regardless, strain- and temperature- dependent outcomes suggest that environmental symbiont acquisition could mitigate the effects of thermal stress on host populations. It is often assumed that vertical transmission of a beneficial symbiont from parent to offspring is the optimal strategy, but our results suggest that environmental acquisition could offer unique benefits under changing conditions.

## Introduction

Many animal hosts rely on microbial symbionts for nutrient supplementation, parasite protection, insecticide resistance, or other services [1–4]. The outcomes of these relationships are often context dependent, with symbiont derived benefits varying based on biotic or abiotic conditions [5, 6]. Such context dependence influences the ecological and evolutionary dynamics of symbiosis and affects our ability to predict the outcomes of symbiotic interactions. Temperature is an important context that affects host-microbe relationships and is particularly relevant considering climate change.

Previous research on temperature dependence in symbiotic outcomes has largely focused on hosts with vertically inherited symbionts. Vertical transmission guarantees symbiont acquisition but comes with two tradeoffs. First, offspring are limited to the same symbiont their mother possessed. Second, symbionts often become highly specialized in their functionality due to genome reduction, a phenomenon in which the symbiont genome shrinks over evolutionary time because life in the stable host environment renders many genes superfluous [7]. This relaxed selection allows deleterious mutations to persist, eventually disabling, then deleting all non-essential genes [8]. Therefore, vertically inherited symbiont genomes lack many genes considered essential in free-living bacteria, including heat shock genes which stabilize proteins and repair DNA under thermal stress [9].

When symbionts lose these thermal stress coping mechanisms, hosts often experience performance declines at high temperatures. Therefore, symbionts are often termed the “Achilles’ heels” of host thermal sensitivity [10–12]. For example, in *Nezara viridula* stinkbugs reared between 25–35°C, their vertically transmitted symbiont’s titer decreased as temperature increased. Consequently, host body size decreased, while development time and mortality increased [10]. Similarly, in pea aphids (*Acyrthosiphon pisum*), brief exposure (3–4 h) to high temperatures (35–38°C) lowered the titer of their vertically transmitted symbiont, triggering decreased host fertility, slowed growth, and increased mortality [12–14]. These systems and others suggest that obligate vertically transmitted symbionts have a lower tolerance to high temperatures than their hosts, thereby restricting host performance under thermal stress [15].

Despite symbiont sensitivity to thermal stress, vertically transmitted symbionts *can* still adapt to high temperatures. Aphids in arid regions have a higher thermal threshold than aphids found in cooler regions [11, 16, 17], due to a symbiont point mutation in heat shock gene *IbpA* that increases the gene’s expression and improves host survival under thermal stress [12, 13]. This demonstrates that symbiont heat shock genes influence temperature-dependent symbiotic outcomes and suggests that host survival increases when symbionts adapt to high temperatures. However, adaptation in vertically transmitted symbionts is limited by their reduced genomes, population bottlenecks, and that selection only acts indirectly on them via effects on the host.

Not all hosts vertically transmit their symbiont, nor are they restricted to a single partner. Some horizontally acquire symbionts directly from conspecifics, as in social insects via oral-oral or oral-anal feeding [18, 19]. Others acquire their partners indirectly from conspecifics (e.g. from symbiont cells secreted into the environment, as in the bobtail squid) [20]. Yet others acquire free-living microbes directly from the environment [21–23]. Hosts that depend on environmental acquisition risk associating with inferior microbes, or not acquiring any symbiont; however, they can potentially select from a diverse pool of partners with different functional capabilities.

Research in horizontally transmitted systems indicates hosts’ thermal niches are less constrained by their symbiont. For example, bumblebee gut microbiota include both bacteria that are transmitted between nestmates and acquired from pollen [24, 25]. These bacteria can survive at higher temperatures than their host, indicating that in some horizontally transmitted symbioses the host is the limiting factor in thermal tolerance, not the symbiont [25]. Similar mechanisms have been proposed in corals that utilize horizontally acquired algal symbionts [26]. After high-temperature events, coral symbionts may be depleted or eliminated entirely [27] and abundances of preferred symbionts in the local environment also decrease [28]. Therefore, corals must replace their symbiont with new strains to survive, with those acquiring thermally tolerant algal species gaining improved performance under thermal stress [29]. These results suggest that symbiont replacement can serve to buffer both terrestrial and marine hosts from increased temperatures.

Like bees and some corals, thousands of insects from the superfamilies Coreoidea, Lygaeoidea, and Pyrrhocoroidea (Hemiptera: Heteroptera: Pentatomomorpha) depend on horizontally acquired *Caballeronia* bacteria [30–32]. *Caballeronia* is required for proper development, with aposymbiotic individuals suffering high mortality, developmental delays, and reduced body size [21, 33–35]. *Caballeronia* must be found and ingested by young nymphs during their second instar (intermolt period) [23, 34]. It is acquired primarily from soil [36], although some species can acquire *Caballeronia* indirectly from conspecifics via cells excreted in frass [35, 37]. The symbiont resides in a specialized section of the midgut called the M4, which seals off after *Caballeronia* acquisition, locking individual insects into a life-long partnership with their initially colonizing strain(s) [30, 36]. The symbiont utilizes host-provided sugars, fatty acids, and nitrogenous wastes and provides the host with essential amino acids and B-vitamins [38].

These insects appear to associate promiscuously with the diverse *Caballeronia* genus [34, 35, 39], with no host-symbiont specialization at insect population or species levels [40]. It is assumed that these insects do not select particular partners; instead, hosted *Caballeronia* strain composition depends on local environmental abundances [35, 39] and competitive interactions among symbiont strains in the host gut [34, 41]. Many strains confer equivalent host benefits under standard laboratory conditions [33, 40], although some consistently confer reduced benefits across host species [33]. In the wild, strain abundances of *Caballeronia* vary geographically [35, 39, 40, 42] and, as would be expected for free-living bacteria, *Caballeronia* can rapidly adapt to local conditions: for example, in soils heavily sprayed with the insecticide fenitrothion, strains evolve to degrade the insecticide [2]. When these strains are ingested, the host gains fenitrothion resistance [2]. This empirically demonstrates that environmentally acquired symbionts can provide locally relevant, context dependent functions to their hosts. We ask whether a similar phenomenon could alter the thermal resilience of an insect host under thermal stress.

As a first step towards answering this question, we evaluated whether hosts perform better under thermal stress when their symbiont has a high thermal optimum. Over 2000 individual Eastern leaf-footed bugs (*Leptoglossus phyllopus,* Coreidae) were provided with one of six phylogenetically distinct *Caballeronia* strains with varying thermal optima and reared at temperatures ranging from 24–40°C. We measured insect development time, mortality, and adult weight as metrics of host performance and *in vivo* symbiont titer as a symbiont performance metric. Our main, overarching hypothesis was that there would be strain-specific tradeoffs in insect outcomes at different temperatures. If true, we made follow-up predictions regarding the mechanism: first, that a symbiont strain’s *in vitro* thermal optimum would predict the degree of benefit it conferred to the host at different temperatures, such that an insect’s performance would be highest when its rearing temperature matched the symbiont’s thermal optimum. Second, we predicted that differences in host outcomes would be driven by symbiont titer, and/or by differences in the symbionts’ heat shock associated genes. We confirmed that host outcomes varied with both symbiont identity and temperature, but were unable to predict these differences based on the symbionts’ thermal optima, symbiont titer, or heat shock gene complements. Contrary to the general assumption that vertical transmission of symbionts from mother to offspring is optimal, our results suggest that intergenerational partner switching, and the acquisition of new phenotypically distinct symbionts, could offer unique benefits to hosts under changing environmental conditions.

## Materials and methods

### Strain collection and identification

Sixty-seven *Burkholderiaceae* symbionts were isolated from coreids following previously published methods [43] with insects collected from Arizona (33 isolates), California (25), Georgia (3), North Carolina (1), and Texas (5) (Table S1).

Symbiont strains were identified using universal 10F/1502R primers (5′- AGTTTGATCATGGCTCAGATTG-3′, 5′- GTTACGACTTCACCCCAG-3′) [44] to sequence a ~ 1530 bp region of the 16S rRNA gene. A phylogeny was generated by aligning sequences using MAFFT v7.450 [45] with default settings and a maximum-likelihood phylogenetic tree was constructed with the GTR + Gamma model of nucleotide substitution using RAxML v8.2.10 [46]. Node support values were calculated using 500 rounds of rapid bootstrapping.

### Evaluating strain thermal performance *in vitro*

Thermal optima for 45 phylogenetically distinct strains were determined by generating growth curves for each strain at four-degree increments from 20–36°C. The final six strains were also evaluated at 40°C. Cultures were diluted to an optical density (OD) of 0.1 after growing overnight at room temperature. The diluted culture was added in triplicate to 96 well plates with sterile yeast extract and glucose (YG) broth as a control [30]. Optical density was measured every 5 min for 48h (Cerillo Stratus plate reader; Charlottesville, VA). Plates were shaken at 270 rpm and temperature was held constant using an Excella E24 Incubator-Shaker (New Brunswick Scientific, Edison, NJ). We completed at least three replicates per strain at each temperature.

We used rolling regressions [47] to calculate the maximum exponential growth rate for each strain because some strains were in log growth phase at the first reading, whereas others never achieved stationary phase. Specifically, we performed linear regressions of log(OD_600_) versus time for a sliding window of six points (30 min) across the entire curve. The 30 min window with the steepest slope was used to estimate maximum growth rate. Estimates for each strain’s replicates were averaged.

### Insect colony and experimental rearing procedure

A laboratory colony of *L. phyllopus* was maintained and housed within a VIVOSUN S108 Grow Tent (Ontario, CA), at 28°C with a 16 L:8D light cycle. The colony contained bush bean plants *(Phaseolus vulgaris*), with raw peanuts for food.

*L. phyllopus* eggs were collected from the colony and moved into screened plexiglass cages, containing 2-week-old cowpea cutting *(Vigna unguiculata*), a cotton-stoppered vial with 0.5% vitamin C water, and two raw peanuts. Water for plants was replaced daily, water vials weekly, and peanuts every two weeks.

At second instar, one of six *Caballeronia* suspensions was added to the cages and the plant and vitamin C were removed. Suspensions contained 0.5% vitamin C with *Caballeronia* grown on YG plates and diluted to approximately OD 1.0. Suspensions were replaced every 24 h for 4 days. Afterwards, the suspension was removed and the plant and vitamin C were returned. Eggs and nymphs up to the third instar were maintained in Percival Scientific environmental chambers (Iowa, USA) set to 28°C, 16 L:8D light cycle, and averaging 50% RH.

Temperature could affect the symbiosis during both the establishment phase (e.g. via effects on environmental abundance of different strains, or through temperature-induced changes in nymph physiology or symbiont-seeking behavior) and during the maintenance phase (e.g. via effects on nutrient provisions to the host after symbiont colonization). We chose to investigate the effect of temperature on the maintenance phase. Temperature treatments therefore began at third instar, after the symbionts colonized the M4, thus isolating the effect of temperature on symbiont function post-establishment. Experimental cages containing an average of four nymphs were placed into Percival chambers set at 20, 24, 28, 32, 36, or 40°C with a 16 L:8D light cycle. Nymphs were reared to adulthood, recording the insects’ stage of development and whether any had died every 1–2 days. Once reaching adulthood, insects were weighed, sexed, and frozen at −80°C. If insects took 60 days or longer to develop from third instar to adult, they were assumed aposymbiotic and excluded from analyses.

### Bacterial Titer analysis

To measure *in vivo Caballeronia* titers across host developmental stages, we followed the previous methods to generate another cohort of insects at temperatures ranging from 24–36°C, collecting the nymphs within 24 h of molting to third, fourth, or fifth instar. Data for adult insects was collected by using a subset of adults from the main experiment.

Genomic DNA was extracted following the insect protocol for Qiagen’s DNeasy Blood & Tissue kit (Germany). Whole body extractions were performed for third and fourth instar nymphs, but only abdomens were used for fifth instars and adults due to kit size limitations.

Symbiont titer was quantified with qPCR using *Caballeronia* specific primers targeting the *dnaA* gene (bsDnaA_F/bsDnaA_R; 5’-AGCGCGAGATCAGACGGTCGTCGATGAT-3′, 5’-TCCGGCAAGTCGCGCACGCA-3′), which has a single copy in the symbiont genome [48]. Standard curves were generated using this primer set on an overnight *Caballeronia* culture followed by serial dilutions to produce concentrations ranging from 10^9^ to 10^1^ copies per μL. To quantify *Caballeronia*, we used PowerUp SYBR Green Master Mix (ThermoFisher Scientific, Massachusetts, USA) following the manufacturer’s protocol with an annealing temperature of 68°C. Samples were run in duplicate on an Applied Biosystems 7300 Real-Time PCR System (ThermoFisher Scientific) with each well containing 4 μL of DNA, 10 μL of Master Mix, 2 μL of each primer (5 μM), and 2 μL of PCR water.

### Comparison of heat shock associated genes

Heat shock genes were identified in the six focal strains via the Integrated Microbial Genomes & Microbiomes database (img.jgi.doe.gov) using KEGG functional group IDs [49]. Identified genes were aligned with MAFFT v7.450 using the default settings and sequences were compared among isolates for each gene (accessions: LP003 - GCA_031450985, LZ003 - GCA_031451465, LZ008 - GCA_031451435, LZ019 - GCA_031450825, LZ062 - GCA_031450785, TF1N1 - GCA_022878925).

### Statistical analysis

Insect survival, development time, fresh adult weight, and symbiont titer were compared across treatments using linear mixed effect models (functions = “lmer”, “glmer”; package = “lme4”) [50]. All models included a random effect for the experimental rearing cages. As an organism’s response to temperature usually follows a thermal performance curve, with peak fitness observed at the thermal optimum and declining at the low and high extremes, we included a quadratic temperature term in all models [51, 52]. We used backwards model selection to find the best-fit fixed effects structure starting with full models that included the interaction between strain identity and temperature, and the interaction between strain identity and a quadratic temperature term. Differences among strains at each temperature were evaluated using “emmeans” (package = “emmeans”) [53] followed by Tukey’s HSD test.

Adult weight differences were evaluated using a Gaussian distribution with sex included in the model because female *Leptoglossus* are larger than males [33]. Development time differences were evaluated with a Poisson distribution. Differences in survival to adulthood were evaluated with binomial error using a logit link function, with an individual surviving to adulthood considered a success and death as a nymph a failure.

Because the 20°C treatment inhibited insect development beyond third instar (based on a pilot with strains R-LZ008 and V-LZ003), and the 40°C treatment resulted in 100% mortality, we concluded that these two temperatures exceed the host’s thermal extremes and these treatments were excluded from all analyses.

Two linear models were used to determine whether there were differences in *in vivo* symbiont titer among strains. The first model evaluated the effects of symbiont strain, host age, and temperature on titer, whereas the second model only evaluated adult insects. These models excluded random effects because the insects for each treatment were reared in the same cage. An R-LP003 adult (28°C) and a V-TF1N1 fifth instar (32°C) were removed from the analysis as both had less than 100 copies of the *Caballeronia dnaA* gene (i.e. < 100 bacterial cells) and were considered aposymbiotic. All analyses were conducted in R v.4.2.2 [54].

## Results

### Symbiont *in vitro* temperature ranges

**Figure 1:**
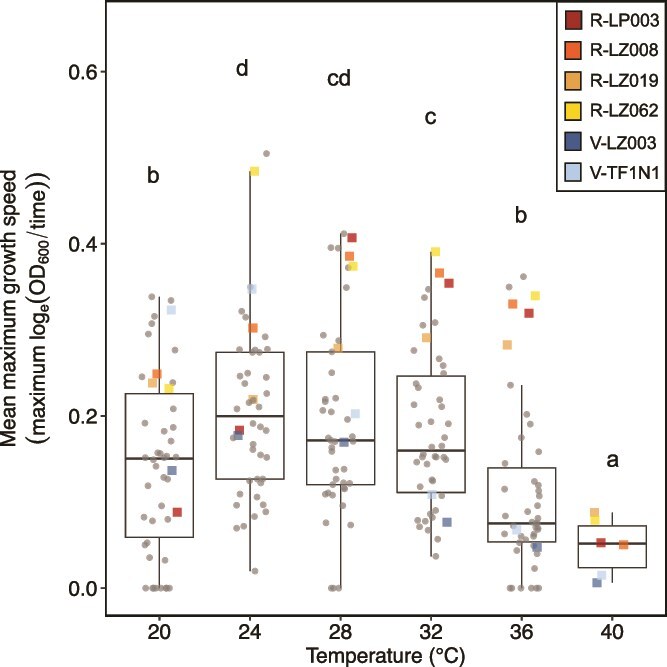
**Maximum growth speeds for the 45 tested strains.** The six strains chosen for subsequent analyses are color coded.

We determined the thermal optima of 45 phylogenetically distinct *Caballeronia* strains (Fig. 1, S1). On average, strains grew best between 24–28°C (*F_5,893_ =* 31.2, *P* value <0.001), intersecting the optimal host range (28–32°C). Growth speed slowed as temperatures decreased towards 20°C or increased towards 36°C. For the subset grown at 40°C, 4/6 strains survived, but growth rate was severely impacted. Overall, 30 strains were considered heat-resistant, growing at all temperatures, and 15 strains were classified as heat-vulnerable, growing poorly at high temperatures or unable to grow at 36°C. Resistant and vulnerable strains were collected from the same locations in similar proportions, with 21 strains collected from Southern California (15 resistant, six vulnerable); 21 from Tucson, Arizona (14 resistant, seven vulnerable); 2 from Northern Texas (1 resistant, 1 vulnerable); and 1 from North Carolina (1 vulnerable).

**Figure 2:**
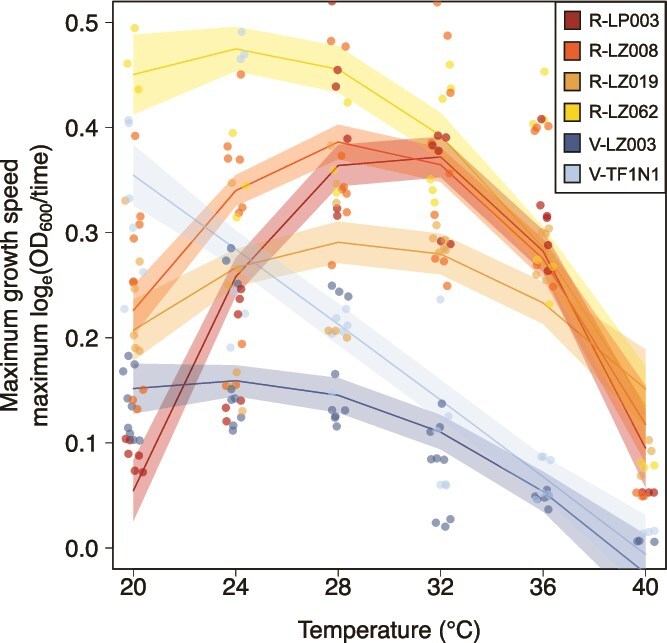
**Thermal growth curves for the six selected strains**. 95% confidence intervals derived from model-based predictions are overlaid with raw data points.

Six focal *Caballeronia* strains were selected for the *in vivo* temperature-performance assays. Focal strains are denoted with “R” if they are heat-resistant (e.g. R-LZ008) or “V” if they are heat-vulnerable (e.g. V-LZ003). We selected V-LZ003 and V-TF1N1 as our heat-vulnerable isolates because they grew poorly at 36°C and did not grow at 40°C. We selected R-LP003, R-LZ008, R-LZ019, and R-LZ062 as our heat-resistant isolates. These isolates grew at 40°C, but their maximum growth speeds trended towards zero (Fig. 2, Table 1).

**Table 1:** Collection locations and annual average summer high temperatures for each symbiont strain used in insect rearing experiments.

Strain	Collection location	Average summer high	Thermal designation
LP003	Texas	33.7 °C	Resistant
LZ003	Arizona	37.8 °C	Vulnerable
LZ008	Arizona	37.8 °C	Resistant
LZ019	Arizona	37.8 °C	Resistant
LZ062	Arizona	37.8 °C	Resistant
TF1N1	North Carolina	31.7 °C	Vulnerable

### Insect performance

**Figure 3:**
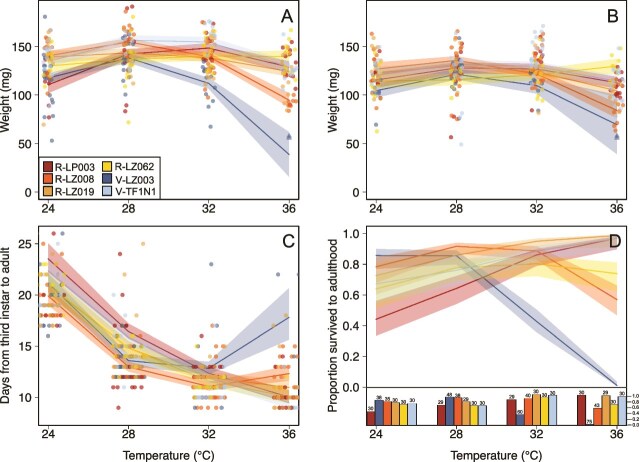
**Performance variation between strains.** 95% confidence intervals derived from model-based predictions overlaid with raw data for (a) female weight, (b) male weight, (c) development time, and (d) survival to adulthood. For weight plots, insects with strain LZ003 at 36°C are indicated by triangles as they had no reported sex and were not included in the model. For the host survival bar plots, numbers above bars indicate the total number of insects reared.

Females were on average 1.14 times larger than males (df = 1, LRT = 88.22, *P* value <0.001). Adult weight also varied by symbiont strain and the rearing temperature (strain:temperature^2^ interaction term: df = 5, LRT = 20.70, *P* value <0.001). Insect weight followed a quadratic relationship with temperature, increasing from 24 to 28°C, then decreasing as temperature increased (Fig. 3A, B, S3a, Table S2). Insects provided with V-LZ003 experienced a severe decline in weight starting at 32°C, being 21% lighter than an average insect colonized by any other strain (except R-LZ008) at that temperature, and 53% smaller than average at 36°C. Insects provided with R-LZ008 also suffered reduced performance at 36°C, being 29% lighter than an average insect provided with any other strain (besides V-LZ003). The final model could not evaluate V-LZ003 at 36°C as only two insects survived to adulthood and both failed to molt properly, preventing sex identification. Despite this, these individuals lie within the 95% confidence intervals at 36°C for both males and females (Fig. 3A, B). Overall, we observed tradeoffs in the weight gain conferred by strains at lower versus higher temperatures. At 24°C R-LZ008 conferred the highest weight, but at 36°C R-LZ008 hosts were the second lightest (Fig. 3A, B; Table S2). In contrast, R-LP003 was the second worst strain at 24°C, but second best at 32°C and 36°C.

Development time from third instar to adulthood also depended on both the symbiont strain and the rearing temperature (strain:temperature^2^ interaction term: df = 5, LRT = 17.24, *P* value <0.01). Insects developed faster regardless of symbiont identity as the temperature increased, but gains in development slowed as the temperature approached 32°C (Fig. 3C, S3b, Table S3). There were tradeoffs among strains in host development speed at different temperatures. Insects colonized with V-TF1N1 developed slowest at cooler temperatures, but fastest at high temperatures, whereas R-LZ008 showed the opposite pattern, being most beneficial at cool temperatures but the second worst at 36°C (Fig. 3C, Table S2). Insects with V-LZ003 showed markedly slowed development time from 32°C to 36°C, taking 8 days (1.8 times) longer to develop to adulthood compared to the average insect provided with any other strain.

The most severe tradeoffs among strains occurred in host survival at different temperatures (strain:temperature^2^ interaction term: df = 5, LRT = 15.20, *P* value = 0.01). For three of the strains, more nymphs survived to adulthood as the temperature increased to 36°C, whereas nymphs inoculated with the other three strains experienced increased mortality at higher temperatures (Fig. 3D, S3c, Table S4). Specifically, insects with R-LZ008 experienced increasing mortality at 32°C but did not differ from the other strains until 36°C, where 58% survived compared to the three high performing strains which averaged 96% survival at 36°C. Hosts with R-LZ062 also experienced greater mortality than the three high performing strains at 36°C, with 70% surviving. Finally, there was a severe decline in survival for V-LZ003 hosting insects with 37% surviving at 32°C, and only 3% at 36°C. From 24°C to 36°C, V-LZ003 and R-LP003 switched their relative rankings, with V-LZ003 conferring the highest survival at cool temperature but the lowest at high temperature, whereas R-LP003 conferred the lowest survival at cool temperatures but the highest at 36°C (Fig. 3D, Table S2). Similarly, R-LZ008 conferred the second highest survival rate at 24°C but the second lowest at 36°C.

### Symbiont titer

**Figure 4:**
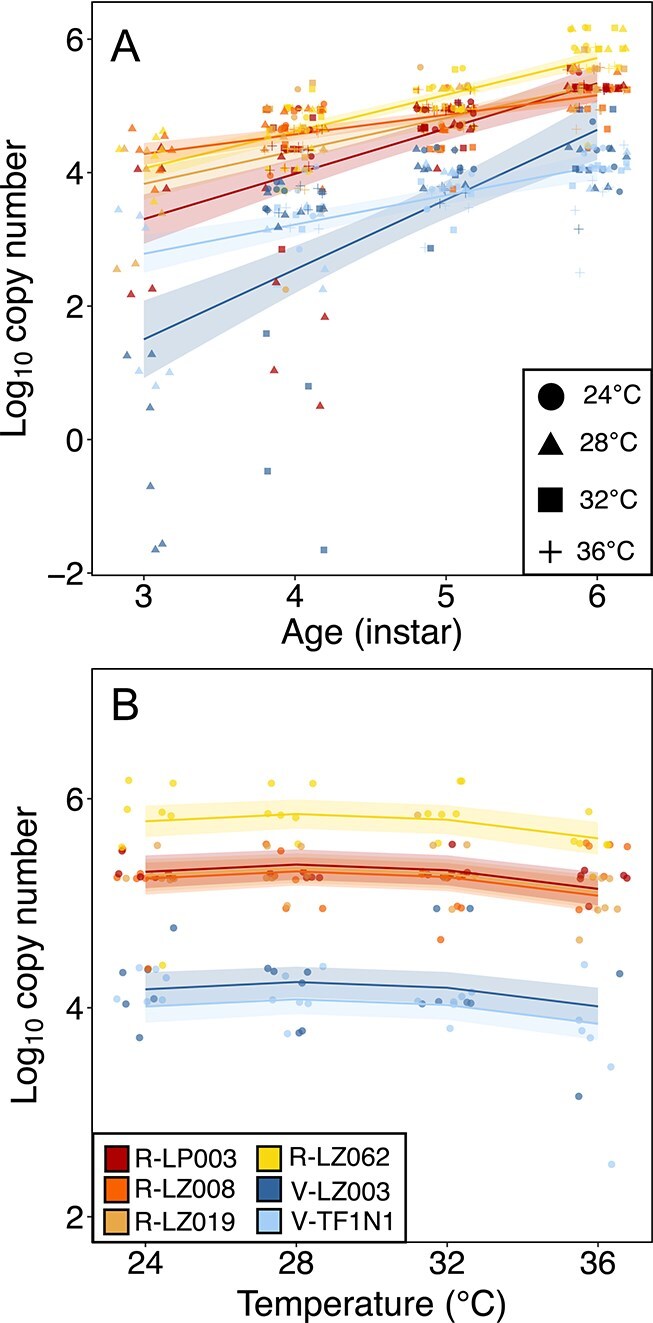
**Titer differences between strains.** 95% confidence intervals derived from model-based predictions (a) across development stages regardless of temperature and (b) across temperatures during the adult life stage. For differences across development time, different shapes indicate the different temperature treatments.

The strains’ *in vivo* titers responded differently to temperature (strain:temperature^2^ interaction term: df = 5, RSS = 192.66, *P* value <0.001) and insect age (strain:age interaction term: df = 5, RSS = 198.98, *P* value <0.001). For all strains, symbiont titer increased as nymphs developed, with R-LZ008 experiencing the slowest growth rate with an increase in copy number of 22% from third instar to adult, and V-LZ003 experiencing the greatest increase of 2825% (Fig. 4A, S3d, Table S5). At any given age and temperature, the *in vivo* titers of the heat-vulnerable strains were 1–2 orders of magnitude lower than the titers of the heat-resistant strains (Fig. 4A).

Because symbiont titer was correlated with host age, we only used adult insect titer data to test our hypothesis that an increase in temperature would lead to a decrease in symbiont titer for vulnerable strains. We found that there were large titer differences among symbiont strains (strain term: df = 5, RSS = 70.91, *P* value <0.001), but titer remained relatively constant across temperatures for each strain (temperature^2^ term: df = 1, RSS = 14.06, *P* value = 0.02) with titer slightly increasing to 28°C, then decreasing as temperature increased (Fig. 4B, Table S6). Insects provided with heat-vulnerable strains had an average titer 1.91–1.98 (80.95–95.76%) fold lower than the heat-resistant strains. One heat-resistant strain, R-LZ062, had an average titer 7–12% greater than other heat-resistant strains.

### Evaluation of stress associated genes

**Figure 5:**
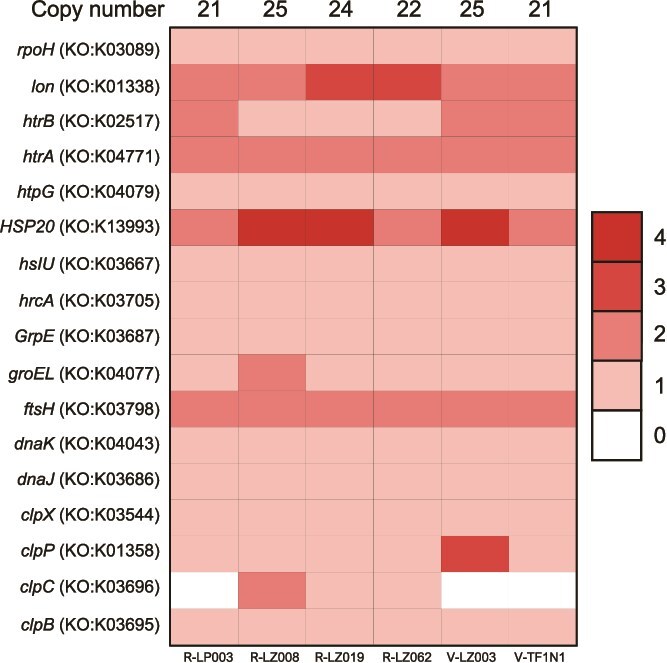
**Heat map of heat shock genes.** Copy number identified by KEGG functional groups. Cell color indicates the number of gene copies detected in the genome.

To attempt to identify genomic mechanisms that may contribute to temperature-dependent differences in host fitness conferred by the symbiont strains, we compared the genomic copy number of heat shock genes across the six *Caballeronia* strains. Of the 23 potential heat shock genes [49], 17 were detected in the focal strains. Some strains possessed multiple copies of these different genes (Fig. 5), with total copy numbers ranging from 21–25. However, no statistical difference was detected in the total number of these genes among strains (*F_5,80_ =* 12.1, *P* value = 0.36).

We also investigated whether our focal symbionts had functional copies of the *CyaA* or *Crp* genes using Protein BLAST [55, 56]. These genes are pivotal in the carbon catabolite repression (CCR) pathway, which switches the bacterium’s metabolism to exploit non-glucose carbon sources under nutrient-deficient and/or high bacterial density conditions. Deactivation of *CyaA* or *Crp* has been shown to improve host outcomes in experimentally evolved *Escherichia coli* symbionts of the Oriental stink bug (*Plautia stali*, Pentatomidae) [57]. If these genes were functional in some *Caballeronia*, they might be correlated with worse host outcomes. However in all six symbionts, both of these genes were absent. More broadly, we found that only 1/152 *Caballeronia* species on NCBI possesses *CyaA* and 18/152 possess *Crp*. This absence is consistent with the deactivation of the CCR pathway promoting symbiosis, as *Caballeronia* are broadly associated with this symbiosis.

## Discussion

We evaluated whether temperature influences the relative benefits conferred to host insects by their environmentally acquired microbial symbiont. We observed tradeoffs between symbionts’ benefits at different temperatures, with strains conferring greater survival, faster development, and larger adult weight at lower temperatures differing from the strains that conferred the best performance at high temperatures. Differences in insect performance and survival were more pronounced at high temperatures, with some strains imposing severe costs (e.g. 97% mortality for V-LZ003 at 36°C, compared to 4% average mortality for the three best strains at 36°C). Consistent with our prediction that matching between symbiont thermal optimum and rearing temperature would determine insect outcomes, insects colonized with heat-vulnerable strain V-LZ003 developed particularly slowly, were very small at adulthood, and suffered high mortality when reared at high temperatures. However, at high temperatures, hosts provided with the other heat-vulnerable strain V-TF1N1 performed well, whereas insects inoculated with heat-resistant R-LZ008 experienced performance declines. Our results demonstrate that host outcomes in this symbiosis are strain- and temperature-dependent and reveal tradeoffs between strain benefits conferred at low versus high temperatures.

To date several bug-*Caballeronia* rearing experiments have evaluated how different symbiont strains affect host insect performance [33, 34, 40]. Excluding a single less-beneficial strain (not included in this study), all *Caballeronia* were found to confer equivalent outcomes to their hosts. However, these insects were reared under optimal conditions between 28 and 30°C. Our findings suggest that although most strains are functionally interchangeable in benign laboratory conditions, strains may not be equivalent under the varying environmental conditions insects experience in the wild. Furthermore, our results may underestimate both the range of *in vitro* temperature optima across the *Caballeronia* genus, and the degree of temperature-dependent variation in host outcomes, because our strains do not represent the entirety of *Caballeronia*’s natural climate range. *Caballeronia* is globally distributed, but our 45 symbiont strains were primarily isolated from insects in central Arizona (21 isolates, including four focal strains) and southern California (21 isolates, 0 focal) which have high average summer temperatures (average daily highs of 35.5°C in CA and 37.8°C in AZ) (weather.gov). The remaining strains were also isolated from areas with warm summers: Texas (five isolates, one focal), Georgia (three isolates, zero focal), and North Carolina (one isolate, one focal). Our symbiont pool was restricted to strains that survived these conditions to be acquired by wild insects. Although our collection locations did not differ in the proportion of heat-resistant versus heat-vulnerable strains, more northern, cooler climates might select for strains that perform better at lower temperatures.

Whether, and to what degree, strain- and temperature-based variation in conferred outcomes is driven by the strains’ thermal optima remains an open question. Overall, we did not observe a strong correlation between the strains’ thermal optima *in vitro* and the temperature at which they conferred maximum benefits *in vivo*. One explanation may be that *Caballeronia*’s thermal optimum in broth culture may differ from that of cells living in an insect host (or in soil). Although the host’s thermoregulatory behaviors should buffer the symbiont from extreme temperatures, evidence suggests the host midgut is a stressful environment for *Caballeronia.* In the M4, the symbiont cells are exposed to antimicrobial peptides (which the host may use to control the symbiont population) and *Caballeronia* produces the stress-associated compound polyhydroxyalkanoate to store energy [38, 58, 59]. Furthermore, nutrient availability is very different within the M4 versus in yeast-glucose broth. The host gut likely provides non-glucose sugars, such as rhamnose and ribose [38], and symbiont cells are likely experiencing high density, starvation conditions [57]. These stressful conditions could alter *Caballeronia’s* temperature response within insects compared to their free-living state.

Maximum growth rate is only one indicator of symbiont performance. Fast growth might be important for initial colonization of the M4, especially when competing with other strains, but after colonization, different traits might be more important to define host benefits. For example, strains that tolerate higher maximum density under a given temperature might be more beneficial (if high symbiont density results in greater nutrient synthesis for hosts). However, our focal strains’ maximum density *in vitro* was no better at predicting host outcomes than maximum growth rate (Fig. S2.) A strain’s synthesis rate of limited nutrients (e.g. vitamins, essential amino acids or cofactors), or its ability utilize substrates provided by the host within the M4, such as diverse sugars or nitrogenous waste products, may also be better predictors of conferred host benefits in the maintenance phase of the relationship. Preliminary exploration of the focal strains’ *in vitro* metabolic abilities suggests that increased ability to metabolize rhamnose and myo-inositol, two carbon compounds which the host provides to *Caballeronia in vivo* [38], might correlate positively with host survival at low temperature and negatively with survival at high temperature (Fig. S4, Fig. S5). Finally, the symbiont’s ability to respond appropriately to the high density, low nutrient conditions of the M4 likely plays a role in host outcomes [57]. Quantifying how temperature affects these abilities in *Caballeronia* may shed light on the mechanisms behind the tradeoffs we observed in symbiont conferred benefits at different temperatures.

### Potential thermal response mechanisms

We evaluated symbiont titer and heat shock gene count as potential mechanisms that could explain strain- and temperature-dependent outcomes in this symbiosis. Across many symbioses, symbiont titer typically decreases under thermal stress, with vertically transmitted symbionts being more sensitive to temperature fluctuations [10, 12]. However, we found temperature had little effect on *Caballeronia* titer, with titers varying much more among strains than across temperatures. The average titer of the heat-resistant strains was almost twice that of the heat-vulnerable strains. We cannot explain this pattern, but perhaps some of the pathways involved in mitigating thermal stress are also used to cope with generally stressful conditions (i.e. host midgut), resulting in a correlation between low titer in the host and poor performance of the free-living bacterium at high temperatures. Unexpectedly, hosts provided with the low titer, heat-vulnerable strain V-TF1N1 performed similarly to hosts with high titer, heat-resistant strains R-LZ019 and R-LP003. This suggests symbiont strains may differ in their optimal *in vivo* titer, perhaps based on the nutrients and other compounds they produce for the host. Alternatively or additionally, the heat-vulnerable strains might be more sensitive to the host’s antimicrobial peptides [59, 60]. Regardless, differences in symbiont titer do not explain temperature-dependent differences in host outcomes.

Our second mechanistic hypothesis was that symbiont survival, and its contribution to improved host performance, under thermal stress increases with the genomic copy number of microbial heat shock associated genes. Instead, all strains had similar gene copy numbers suggesting (i) different strains may express heat shock genes at different levels under thermal stress, (ii) different symbiont species may produce different types of heat shock proteins that we did not identify, or (iii) heat shock genes are not a contributing factor in host performance. To identify the mechanisms that explain strain-by-temperature dependent outcomes, a promising next step could be the investigation of these symbionts’ *in vivo* transcriptional response to different temperatures.

### Implications for host fitness under changing temperature regimes

In environmentally acquired symbioses, the environment will operate as a selective force on free-living symbiont populations, as well as on the host-symbiont holobiont. We propose for bug-*Caballeronia*, temperature will first act as a selective filter on free-living *Caballeronia*. Because high temperatures should select for heat-resistant symbionts and reduce the abundance of heat-vulnerable strains, hosts should be more likely to acquire heat-resistant symbionts in hotter environments. Next, temperature could affect host colonization by changing a nymphs’ physiology or symbiont-seeking behaviors. After colonization, although *Caballeronia* is buffered from extreme temperatures by host behavioral thermoregulation, our findings demonstrate that temperature applies an additional indirect force on symbiotic outcomes*,* presumably via effects on host physiology.

If the host acquires a sub-optimal symbiont, it cannot be replaced during the insect’s lifetime. The symbiotic M4 midgut region seals off within 24 h of initial symbiont acquisition, preventing hosts from acquiring additional strains later in life [61]. However, wild insects often acquire multiple symbiont strains during the ~24 h critical exposure period; one symbiont strain tends to dominate the M4, with one or more additional strains present at lower abundance [42]. It is possible that if a wild insect experiences a change in temperature and possesses a low abundance strain better matched to the new conditions, that strain might increase in abundance within the M4. This would depend both upon the insect acquiring an appropriate strain during the second instar, and on the strain successfully competing against the dominant strain. Since *Caballeronia* colonizes crypts (invaginations of the gut lining) within the M4, the secondary strain would have to overcome a strong priority effect within each crypt [41].

Our study suggests that acquiring free-living microbial symbionts could release hosts from their symbiont’s limitations by facilitating partner switching. This phenomenon has been demonstrated in leaf-cutter ants and bark beetles, which utilize fungal species with varying thermal tolerances to colonize new niches [62, 63]. However, most previous research on temperature dependence in symbiotic outcomes has largely focused on hosts with vertically transmitted symbionts. Although it is often casually assumed that vertical symbiont transmission from parent to offspring is the optimal strategy, vertically transmitted symbionts are often more sensitive to high temperatures than their host and can limit their host’s viable temperature range. Our results contribute to a small but growing literature that suggests that terrestrial environmentally acquired symbioses may be more resilient to changing temperatures than vertically transmitted symbioses.

## Conclusions

We began with the proposition that intergenerational partner switching enabled by environmental symbiont acquisition could result in better host outcomes under changing conditions. Our finding that strains vary in the degree of benefits they confer at different temperatures is a necessary prerequisite. To further evaluate this possibility, next steps will be to test whether strains in the field are adapted to local climate conditions (strain-climate matching over space), whether temperature optima in field *Caballeronia* populations evolve in response to climate (strain-climate matching over time), whether insects in the field perform better when they acquire local strains versus strains from other sites, and whether this effect, if present, is more pronounced for insects transplanted to new conditions versus insects reared in conditions similar to parental generations. Furthermore, we reared both symbionts and insects at constant temperatures, but investigating responses to short-term exposure to temperature stress would be valuable to mirror natural thermal stress events (e.g. heat waves).

Our findings suggest that in the bug-*Caballeronia* symbiosis, host outcomes vary among strains and have temperature-dependent tradeoffs. At high temperature, host performance diverged substantially and symbiont identity mattered more. Ultimately, environmental acquisition can be risky, as there is no guarantee a host will acquire a beneficial symbiont strain, but the resulting ease of intergenerational switching between phenotypically distinct symbionts may offer unique benefits to hosts under changing environmental conditions.

## Supplementary Material

Supplemental_Information_v2_wraf056

Table_S1_wraf056

## Data Availability

The datasets generated and analyzed during the study are available on DRYAD (https://doi.org/10.5061/dryad.zs7h44jjj). Sequences used to build the phylogeny are available on GenBank under accession numbers OQ286222–OQ286289. Symbiont genomes for the focal strains were downloaded from GenBank (Accession numbers: GCA_031450985, GCA_031451465, GCA_031451435, GCA_031450825, GCA_031450785, GCA_022878925).
