# Supplementary material for: Temperature influences outcomes of an environmentally acquired symbiosis
Source: ISME J. 2025 Mar 21;19(1):wraf056. doi: 10.1093/ismejo/wraf056 (PMC11995993; doi:10.1093/ismejo/wraf056)
Supplement: Supplemental_Information_v2_wraf056 [file supplemental_information_v2_wraf056.pdf]

Supplemental Figures

Fig S1. 16S rRNA phylogeny of tested *Burkholderiaceae* strains. The tree was rooted with outgroup *Cupriavidus pauculus* (strain BHJ32i). Select named species were added followed with their accession numbers or strain information. Species names in the phylogenies were color-coded with black indicating species tested for thermal tolerance, and red indicating the strain was used for the rearing experiment. Points to the left of strain names indicate if they were considered heat-vulnerable (blue) or heat-resistant (red). Node support values were calculated using rapid bootstrapping, and nodes with less than 40% support have been collapsed. Scale bar indicates substitutions per site.

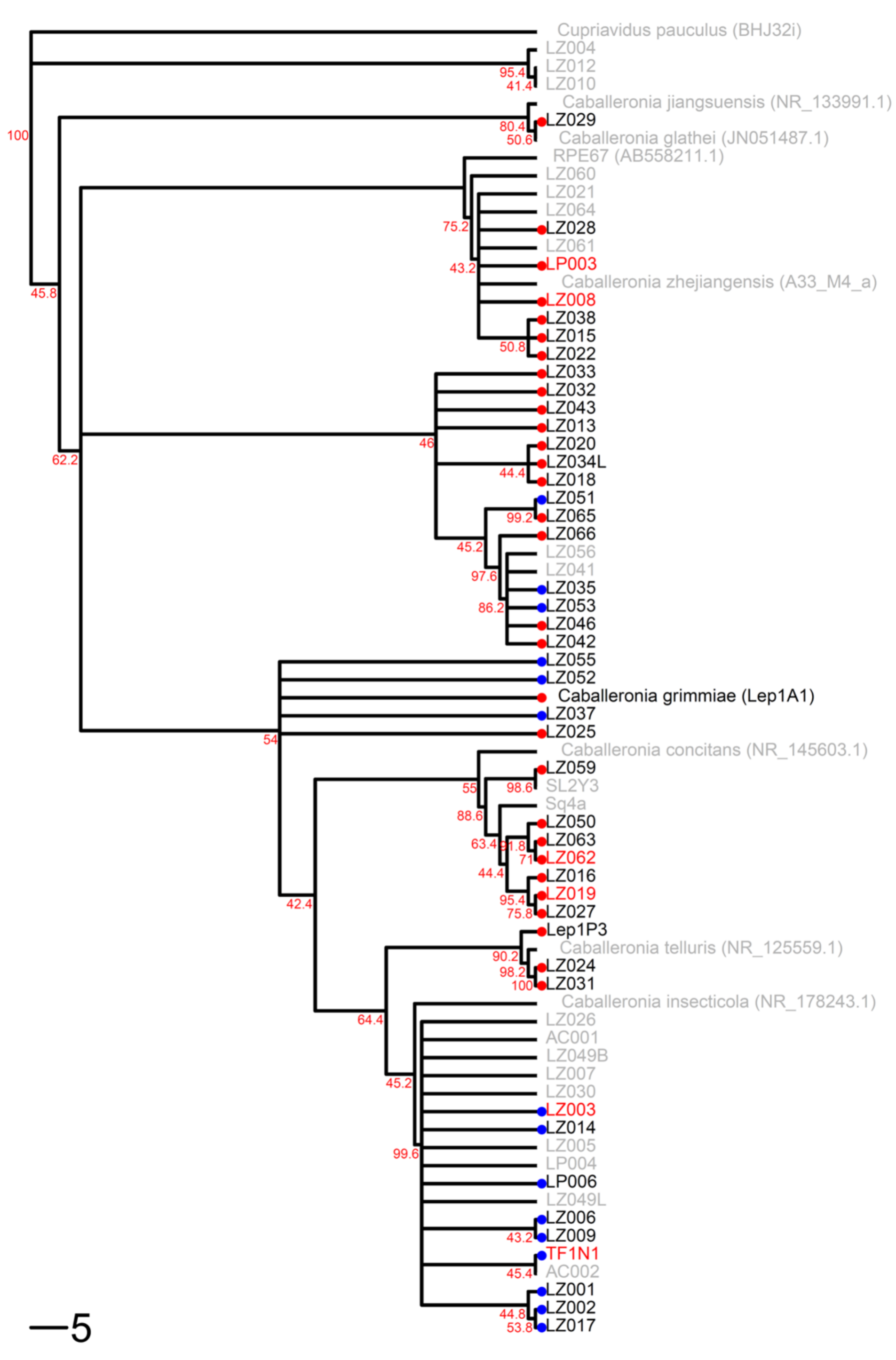
Cupriavidus pauculus (BHJ32i)
LZ004
LZ012
LZ010
Caballeronia jiangsuensis (NR_133991.1)
LZ029
Caballeronia glathei (JN051487.1)
RPE67 (AB558211.1)
LZ060
LZ021
LZ064
LZ028
LZ061
LP003
Caballeronia zhejiangensis (A33_M4_a)
LZ008
LZ038
LZ015
LZ022
LZ033
LZ032
LZ043
LZ013
LZ020
LZ034L
LZ018
LZ051
LZ065
LZ066
LZ056
LZ041
LZ035
LZ053
LZ046
LZ042
LZ055
LZ052
Caballeronia grimmiae (Lep1A1)
LZ037
LZ025
Caballeronia concitans (NR_145603.1)
LZ059
SL2Y3
Sq4a
LZ050
LZ063
LZ062
LZ016
LZ019
LZ027
Lep1P3
Caballeronia telluris (NR_125559.1)
LZ024
LZ031
Caballeronia insecticola (NR_178243.1)
LZ026
AC001
LZ049B
LZ007
LZ030
LZ003
LZ014
LZ005
LP004
LP006
LZ049L
LZ006
LZ009
TF1N1
AC002
LZ001
LZ002
LZ017
5

Fig S2. Maximum OD_{600} curves for the six selected strains. Maximum OD decreased as temperature increased for most isolates; the exceptions was V-LZ003, which exhibited a slight increase in maximum OD from 20 – 24°C but and then decreased from 28 – 40°C. The strains' relative responses to high temperatures were largely comparable between maximum growth speed (Fig 2) and maximum OD, with heat-resistant strains showing both faster growth and higher OD than the heat-vulnerable strains at 36 and 40°C. At lower temperatures the strains tended to be more similar to each other in maximum OD than they were in maximum growth speed. The most notable difference between the metrics was that R-LZ062 was the fastest growing strain from 20 – 28°C but reached some of the lowest maximum ODs at those temperatures. 95% confidence interval derived from model-based predictions overlaid with raw data point. At 36°C, V-LZ003 and V-TF1N1 did not reach stationary phase after 48 h.

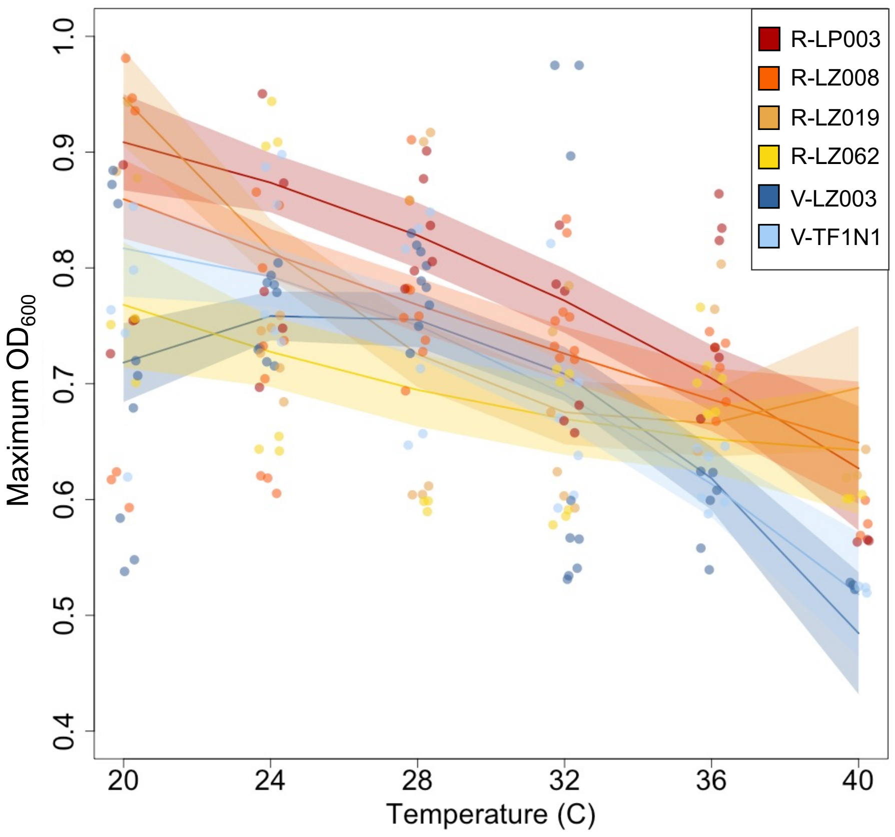

Fig S3. Raw data points for performance variation between strains. (a) host weight with females marked as circles, males as triangles, and unidentified individuals as squares; (b) development time; (c) survival to adulthood; and (d) log(copy number) across temperature and host developmental stages.

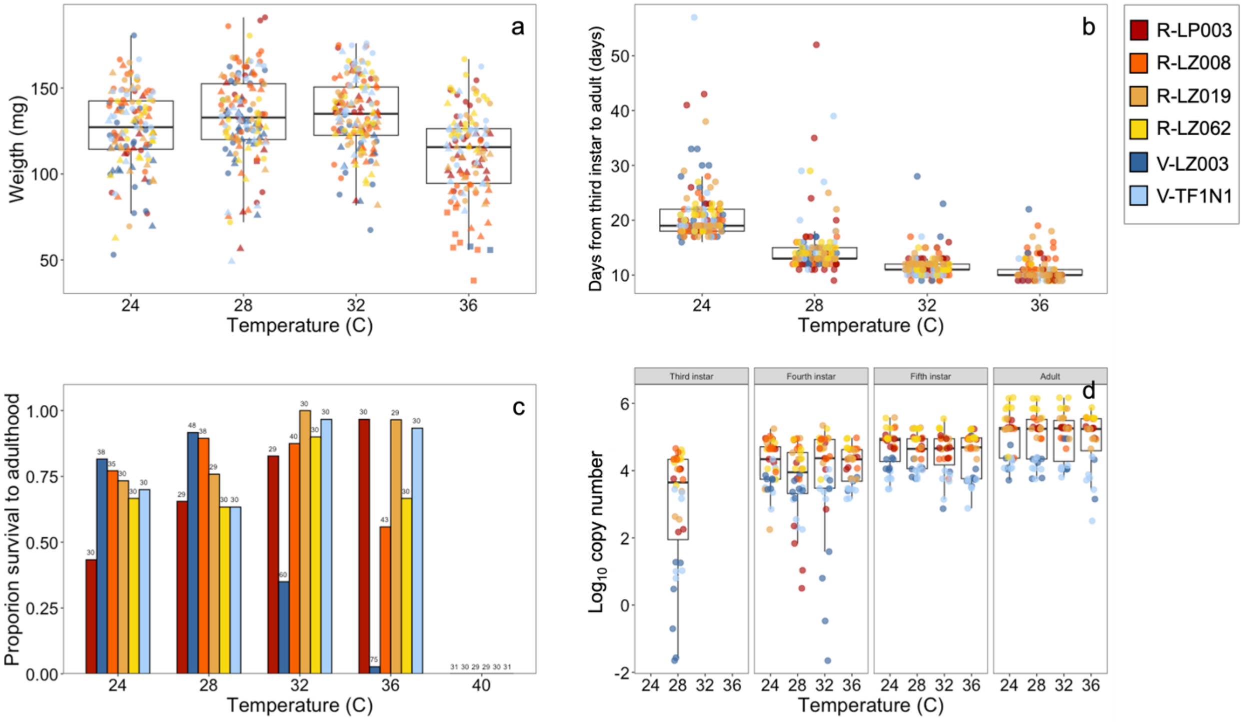

Fig S4. Comparison of symbiont strains' utilization of different types of (a,b) carbon and (c,d) nitrogen compounds. To evaluate possible metabolic differences among the six focal symbiont strains, we used Biolog plates (Biolog, Inc., California, USA) to measure utilization of carbon compounds (plate PM1) and nitrogenous compounds (plate PM3B). On the PM3B plate, glucose was provided as the carbon source at a final concentration of 20 mM. Plates were inoculated with an initial OD of about 0.38 (85% transmittance) and incubated at 28°C for 48 h. Absorbance was read at 590 nM on a Biotek Synergy H1 plate reader. Absorbances were normalized by subtracting the value of the negative control well from all measurements, dividing all measurements by the average absorbance of the plate, and setting negative values to zero. A higher normalized absorbance indicates greater catabolism of the compound.

Panels (a) and (b) show the same data, as do panels (c) and (d), but the isolates are sorted differently on the X-axes: In panels (a) and (c), isolates are sorted in order of increasing host survival probability (from left to right) at 24°C, whereas in panels (b) and (d) isolates are sorted in order of increasing host survival probability at 36°C.

There was no relationship between the survival probability conferred by different *Caballeronia* strains at low (24°C) or high (36°C) temperature and the strains' ability to use different categories of carbon compounds or nitrogen compounds.

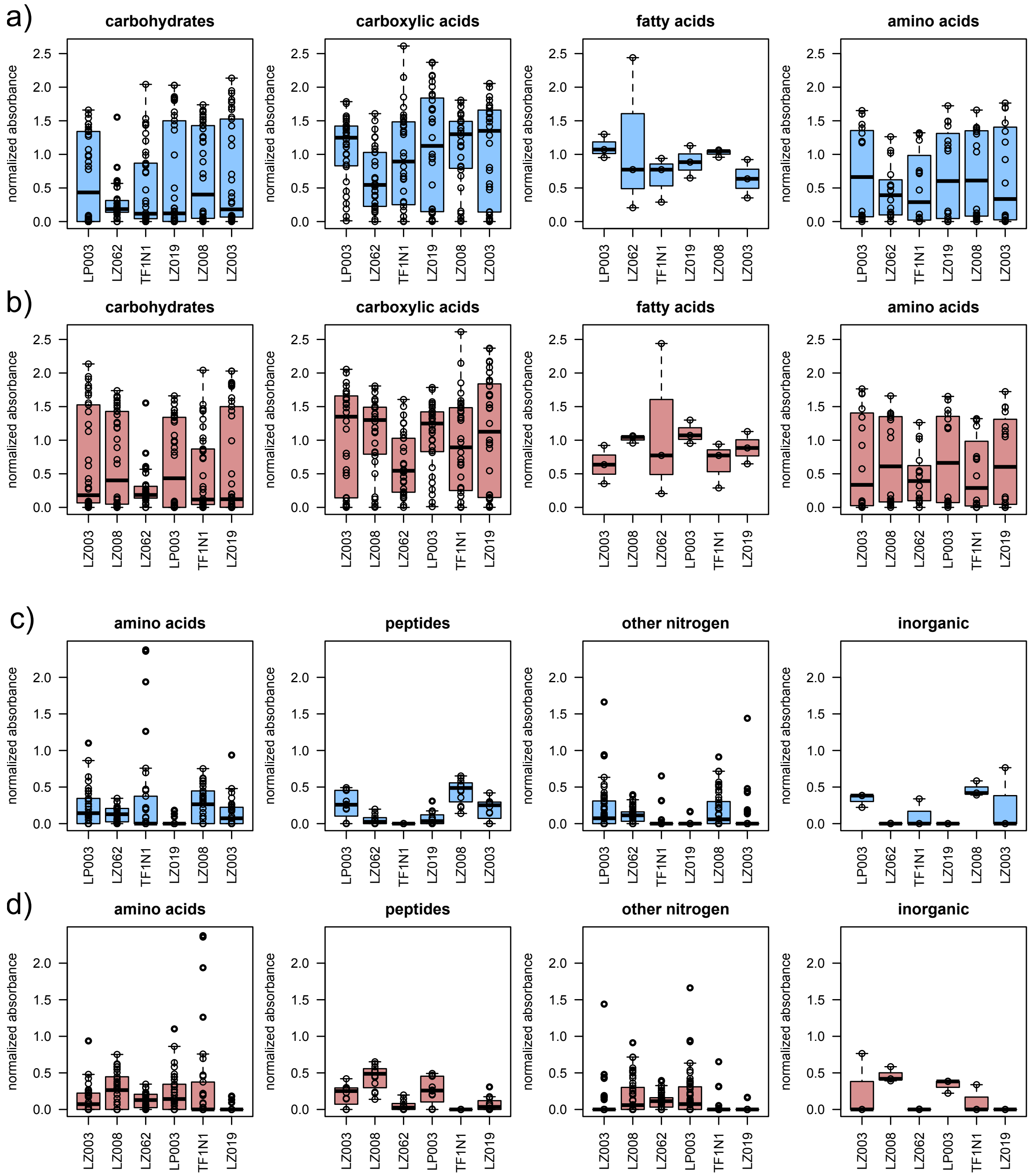

a)
carbohydrates
carboxylic acids
fatty acids
amino acids
normalized absorbance
LP003
LZ062
TF1N1
LZ019
LZ008
LZ003
b)
carbohydrates
carboxylic acids
fatty acids
amino acids
normalized absorbance
LZ003
LZ008
LZ062
LP003
TF1N1
LZ019
c)
amino acids
peptides
other nitrogen
inorganic
normalized absorbance
LP003
LZ062
TF1N1
LZ019
LZ008
LZ003
d)
amino acids
peptides
other nitrogen
inorganic
normalized absorbance
LZ003
LZ008
LZ062
LP003
TF1N1
LZ019

Fig S5. Comparison of symbiont strains' utilization of carbon and nitrogen compounds likely provided by the host *in vivo.* Ohbayashi et al. 2019 concluded that host insects likely provide ribose, rhamnose, myo-inositol, and fatty acids as carbon sources and allantoin and urea as nitrogen sources to *Caballeronia in vivo.* We therefore explored the relationship between the focal isolates' abilities to use these compounds at 28°C, using Biolog assays as described above in Fig S4, and conferred host survival probability at (a) 24°C and (b) 36°C. (Fatty acids are presented in Fig S4.) Note that normalized absorbances for each isolate are the same in panels (a) and (b) but survival probabilities are different.

a)

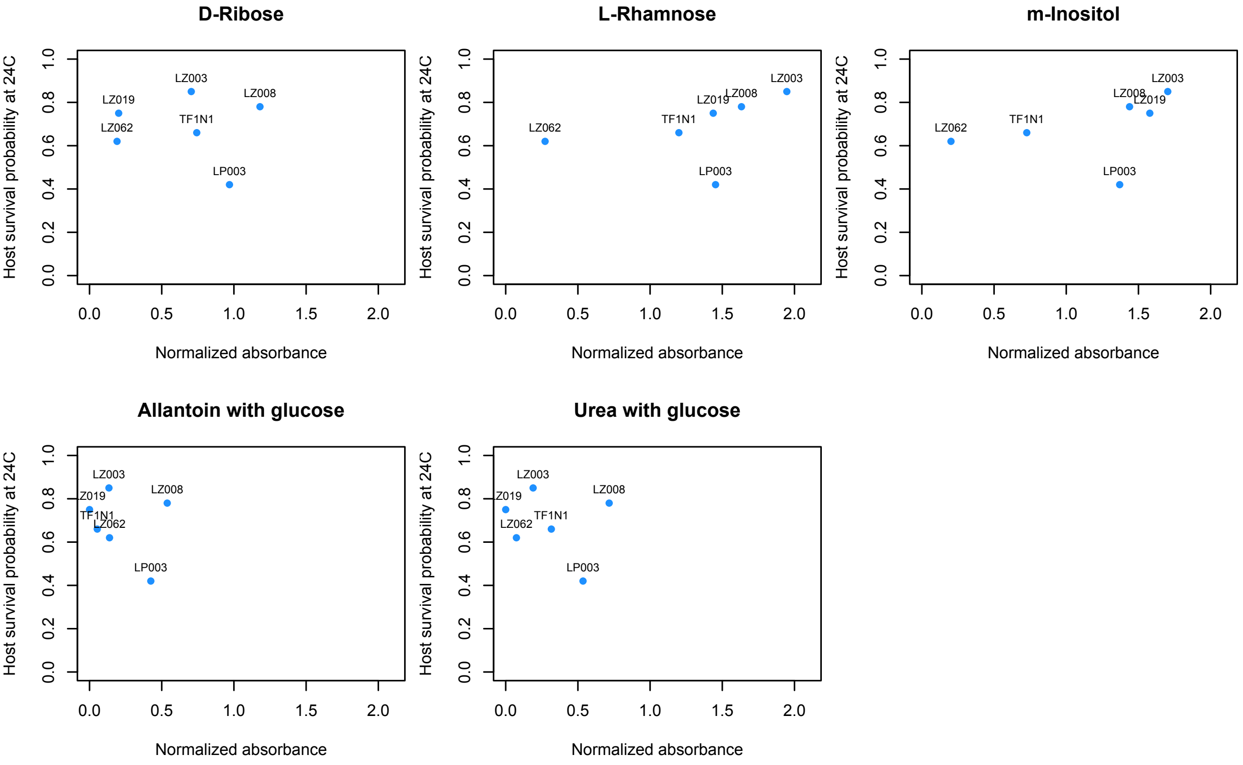

b)

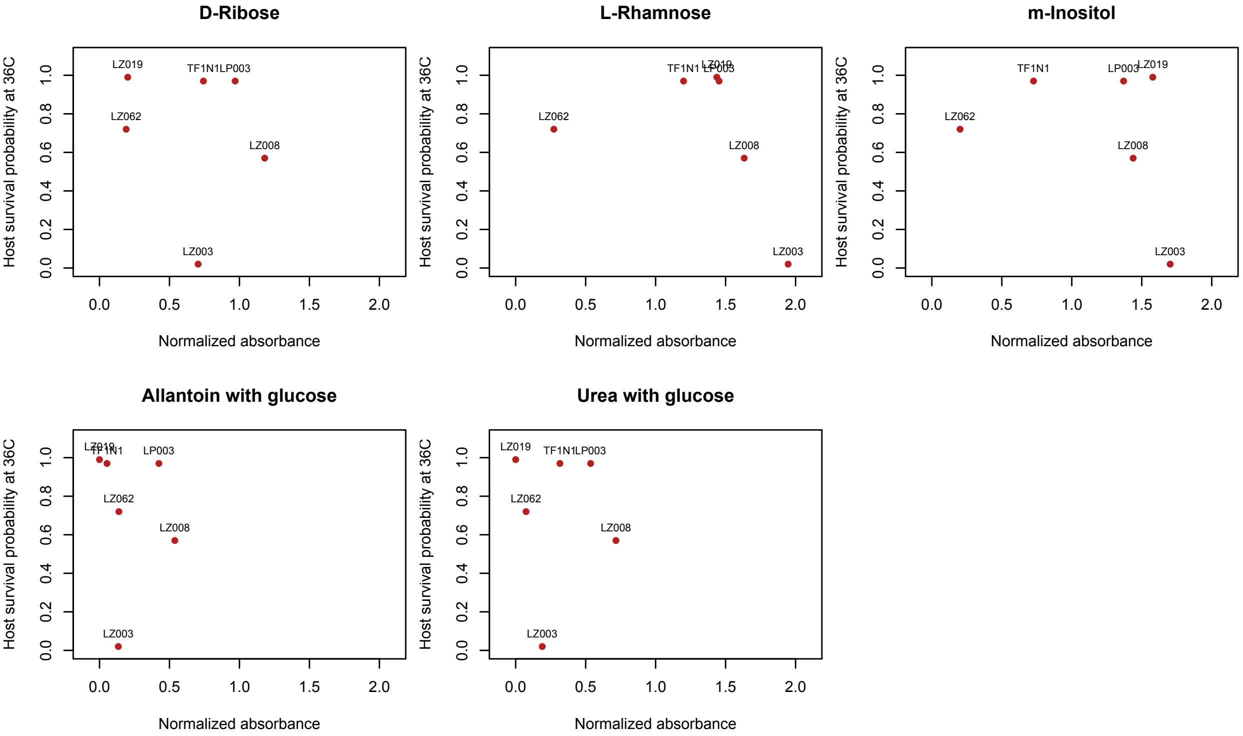
D-Ribose
LZ019
TF1N1
LP003
LZ062
LZ008
LZ003
L-Rhamnose
TF1N1
LZ019
LP003
LZ062
LZ008
LZ003
m-Inositol
TF1N1
LP003
LZ019
LZ062
LZ008
LZ003
Allantoin with glucose
LZ019
TF1N1
LP003
LZ062
LZ008
LZ003
Urea with glucose
LZ019
TF1N1
LP003
LZ062
LZ008
LZ003
Host survival probability at 36C
0.0
0.2
0.4
0.6
0.8
1.0
Normalized absorbance
0.0
0.5
1.0
1.5
2.0

Table S2. Adult insect final weights. V-LZ003 is left blank at 36°C as only 2 insects survived to adulthood, and were excluded from the analysis. The letters denote significant differences between strains within a given temperature group based on the statistical model (model: Weight~Strain:Temperature^2 + Sex; Tukey's HSD < 0.05), while no letter indicates no significant differences.

Temp	Strain	Mean ± SEM
24°C	R-LZ008	135.4779 ± 4.3171 **b**
	V-TF1N1	132.0522 ± 4.7388 **b**
	R-LZ019	131.0589 ± 4.3863 **b**
	R-LZ062	123.2291 ± 4.9189 **ab**
	R-LP003	116.9799 ± 6.0420 **ab**
	V-LZ003	111.4671 ± 4.0469 **a**
28°C	R-LZ008	140.1868 ± 5.1842
	R-LZ019	135.3133 ± 6.7385
	V-TF1N1	131.6996 ± 6.9780
	R-LP003	129.7139 ± 7.2316
	V-LZ003	129.3618 ± 5.1909
	R-LZ062	122.9520 ± 6.9145
32°C	V-TF1N1	150.8546 ± 4.0780 **c**
	R-LP003	141.1066 ± 4.3693 **bc**
	R-LZ008	134.9048 ± 3.4385 **bc**
	R-LZ062	133.1303 ± 4.0473 **bc**
	R-LZ019	132.2482 ± 3.9448 **b**
	V-LZ003	109.6063 ± 4.3236 **a**
36°C	R-LZ062	132.7736 ± 3.8252 **c**
	R-LP003	118.9807 ± 3.5640 **bc**
	V-TF1N1	117.9663 ± 3.5591 **bc**
	R-LZ019	114.2146 ± 3.5493 **b**
	R-LZ008	86.07515 ± 4.6369 **a**
	V-LZ003	

Table S3. Development time to adult from third instar.The letters denote significant differences between strains within a given temperature group based on the statistical model (model: Development~Strain:Temperature^2; Tukey's HSD < 0.05), while no letter indicates no significant differences.

Temp	Strain	Mean ± SEM
24°C	R-LP003	22.7183 ± 1.8924
	V-TF1N1	22.3567 ± 1.6274
	V-LZ003	21.0307 ± 1.2881
	R-LZ062	20.8603 ± 1.5532
	R-LZ019	20.6773 ± 1.5201
	R-LZ008	19.9868 ± 1.2771
28°C	V-TF1N1	17.3684 ± 0.9561 **c**
	R-LP003	16.6842 ± 0.9371 **bc**
	R-LZ062	14.9474 ± 0.8870 **abc**
	R-LZ019	14.6087 ± 0.7970 **abc**
	V-LZ003	13.6410 ± 0.5914 **ab**
	R-LZ008	13.2059 ± 0.6232 **a**
32°C	V-LZ003	12.7619 ± 0.7796
	R-LP003	12.2083 ± 0.7132
	R-LZ019	12.1667 ± 0.6368
	R-LZ062	11.5556 ± 0.6542
	V-TF1N1	11.0000 ± 0.6159
	R-LZ008	10.9143 ± 0.5584
36°C	V-LZ003	18.0000 ± 3.0000 **b**
	R-LZ008	12.4167 ± 0.7193 **ab**
	R-LZ019	10.9286 ± 0.6247 **ab**
	R-LP003	10.1034 ± 0.5902 **a**
	R-LZ062	10.1000 ± 0.7106 **a**
	V-TF1N1	9.7857 ± 0.5912 **a**

Table S4. Proportion of insects surviving to adulthood. The letters denote significant differences between strains within a given temperature group based on the statistical model (model: Survival~Strain:Temperature^2; Tukey's HSD < 0.05), while no letter indicates no significant differences.

Temp	Strain	Mean ± SEM
24°C	V-LZ003	0.8158 ± 0.0629 **b**
	R-LZ008	0.7714 ± 0.0710 **ab**
	R-LZ019	0.7333 ± 0.0807 **ab**
	V-TF1N1	0.7000 ± 0.0837 **ab**
	R-LZ062	0.6667 ± 0.0861 **ab**
	R-LP003	0.4333 ± 0.0905 **a**
28°C	V-LZ003	0.9342 ± 0.0432
	R-LZ008	0.9311 ± 0.0487
	R-LZ019	0.8071 ± 0.1148
	R-LP003	0.6685 ± 0.1473
	R-LZ062	0.6752 ± 0.1444
	V-TF1N1	0.6458 ± 0.1505
32°C	R-LZ019	1.0000 ± 0.0000 **ab**
	V-TF1N1	0.9715 ± 0.0310 **b**
	R-LZ062	0.9107 ± 0.0554 **b**
	R-LZ008	0.8874 ± 0.0533 **b**
	R-LP003	0.8408 ± 0.0763 **b**
	V-LZ003	0.3477 ± 0.0761 **a**
36°C	R-LP003	0.9681 ± 0.0321 **c**
	R-LZ019	0.9669 ± 0.0333 **c**
	V-TF1N1	0.9361 ± 0.0457 **c**
	R-LZ062	0.6743 ± 0.0942 **bc**
	R-LZ008	0.5602 ± 0.0825 **b**
	V-LZ003	0.0255 ± 0.0184 **a**

Table S5. Symbiont titers (loggged copy number) across host lifespan. The letters denote significant differences between strains within a given age group based on the statistical model (model: log(copy number)~Strain:Temperature^2 + Age:Strain; Tukey's HSD < 0.05).

Temp	Strain	Mean ± SEM
3rd	R-LZ008	4.2729 ± 0.3864 **c**
	R-LZ062	4.2727 ± 0.3864 **c**
	R-LZ019	3.5488 ± 0.3864 **bc**
	R-LP003	3.4425 ± 0.2668 **bc**
	V-TF1N1	2.1303 ± 0.3864 **b**
	V-LZ003	-0.1527 ± 0.3864 **a**
4th	R-LZ008	4.6396 ± 0.1627 **d**
	R-LZ062	4.5602 ± 0.1658 **d**
	R-LZ019	4.3997 ± 0.1658 **cd**
	R-LP003	3.8593 ± 0.1658 **bc**
	V-TF1N1	3.4241 ± 0.1596 **ab**
	V-LZ003	2.9999 ± 0.1658 **a**
5th	R-LZ062	5.1155 ± 0.0577 **d**
	R-LZ019	4.8818 ± 0.0577 **cd**
	R-LP003	4.8101 ± 0.0577 **c**
	R-LZ008	4.7415 ± 0.0577 **c**
	V-LZ003	3.9634 ± 0.0630 **b**
	V-TF1N1	3.7100 ± 0.0589 **a**
Adult	R-LZ062	5.7741 ± 0.0622 **c**
	R-LP003	5.2801 ± 0.0650 **b**
	R-LZ019	5.2539 ± 0.0634 **b**
	R-LZ008	5.2165 ± 0.0611**b**
	V-LZ003	4.1433 ± 0.0746 **a**
	V-TF1N1	4.0021 ± 0.0634 **a**

Table S6. Symbiont titers (loggged copy number) for adult insects. The letters denote significant differences between strains within a given temperature group based on the statistical model (model: log(copy number)~Strain + Temperature^2; Tukey's HSD < 0.05).

Temp	Strain	Mean ± SEM
24°C	R-LZ062	5.6963 ± 0.1337 **b**
	R-LP003	5.3027 ± 0.1445 **b**
	R-LZ019	5.2916 ± 0.1445 **b**
	R-LZ008	5.1496 ± 0.1445 **b**
	V-LZ003	4.2172 ± 0.1445 **a**
	V-TF1N1	4.2093 ± 0.1445 **a**
28°C	R-LZ062	5.8969 ± 0.0929 **c**
	R-LZ019	5.3036 ± 0.0929 **b**
	LP003	5.2649 ± 0.1018 **b**
	R-LZ008	5.2424 ± 0.0929 **b**
	V-LZ003	4.1402 ± 0.0929 **a**
	V-TF1N1	4.1038 ± 0.0929 **a**
32°C	R-LZ062	5.9122 ± 0.1108 **c**
	R-LZ019	5.4017 ± 0.1108 **b**
	LP003	5.3246 ± 0.1108 **b**
	R-LZ008	5.1141 ± 0.0960 **b**
	V-LZ003	4.3484 ± 0.1108 **a**
	V-TF1N1	4.0350 ± 0.1108 **a**
36°C	R-LZ062	5.5683 ± 0.1510 **b**
	R-LZ008	5.4034 ± 0.1510 **b**
	LP003	5.2153 ± 0.1510 **b**
	R-LZ019	4.9936 ± 0.1510 **b**
	V-LZ003	3.7393 ± 0.2615 **a**
	V-TF1N1	3.6204 ± 0.1510 **a**
